# Thermal display glove for interacting with virtual reality

**DOI:** 10.1038/s41598-020-68362-y

**Published:** 2020-07-09

**Authors:** Seung-Won Kim, Sung Hee Kim, Choong Sun Kim, Kyoungsoo Yi, Jun-Sik Kim, Byung Jin Cho, Youngsu Cha

**Affiliations:** 1 Center for Medical Robotics, Korea Institute of Science and Technology (KIST), Seoul, 02792 Republic of Korea; 2 Center for Intelligent and Interactive Robotics, Korea Institute of Science and Technology (KIST), Seongbuk-gu, Seoul, 02792 Republic of Korea; 3 School of Electrical Engineering, Korea Advanced Institute of Science and Technology (KAIST), Daejeon, 34141 Republic of Korea; 4 TEGway Co. Ltd., Daejeon, 34325 Republic of Korea; 5 Engineering Research Center (ERC) for Flexible Thermoelectric Device Technology, Daejeon, 34141 Republic of Korea

**Keywords:** Mechanical engineering, Biomedical engineering

## Abstract

Thermal perception is essential for the survival and daily activities of people. Thus, it is desirable to realize thermal feedback stimulation for improving the sense of realism in virtual reality (VR) for users. For thermal stimulus, conventional systems utilize liquid circulation with bulky external sources or thermoelectric devices (TEDs) on rigid structures. However, these systems are difficult to apply to compact wearable gear used for complex hand motions to interact with VR. Furthermore, generating a rapid temperature difference, especially cooling, in response to a thermal stimulus in real-time is challenging for the conventional systems. To overcome this challenge and enhance wearability, we developed an untethered real-time thermal display glove. This glove comprised piezoelectric sensors enabling hand motion sensing and flexible TEDs for bidirectional thermal stimulus on skin. The customized flexible TEDs can decrease the temperature by 10 °C at room temperature in less than 0.5 s. Moreover, they have sufficiently high durability to withstand over 5,000 bends and high flexibility under a bending radius of 20 mm. In a user test with 20 subjects, the correlation between thermal perception and the displayed object’s color was verified, and a survey result showed that the thermal display glove provided realistic and immersive experiences to users when interacting with VR.

## Introduction

People perceive various stimuli from the surrounding environment by using the sensory receptors of the body. This allows them to comprehend their situation and respond according to the surrounding environment, which facilitates survival in nature. With respect to virtual reality (VR) and augmented reality (AR), various types of wearable gear embedding sensors and actuators have been developed to provide stimulus from the virtual or augmented environment to users in the real world^1–3^. To manipulate an object in VR and AR environments, a glove-type device is the most appropriate for the following reasons. A human hand has 27 degrees-of-freedom (DOFs), which is the highest number of DOFs among all body parts, thereby enabling the manipulation of objects with complicated forms. Moreover, according to the cortical homunculus of Penfield^4^, the hand, as an isolated part, is the most sensitive body part with different types of sensory receptors on the skin that enable us to feel multiple sensory modalities, including pressure, vibration, stretch, pain, and temperature. Furthermore, it is possible to perceive a wide range of stimuli ranging from light physical actions such as gentle touching to coarser actions such as pinching.

To realize physical interaction with virtual objects, three major feedback displays have been employed in glove-type devices: tactile, haptic, and thermal^5^. The tactile and haptic feedback displays employ micro motors and piezoelectric actuators, and they provide contact information by applying force and displacement to the mechanoreceptors in the skin of the hand. On the contrary, thermal feedback displays induce heat transfer, and the thermoreceptors in the skin detect the difference in temperature. The perception of thermal change is important with respect to the physiology and survival of human beings^6^. Humans are endotherms, and they maintain their body temperature such that the perception of temperature difference is directly related to survival. Additionally, thermal perception helps humans to avoid extremely high or low temperature environments that would damage the body. Despite this significance of thermal perception, it is difficult to induce real-time thermal change in glove-type devices due to the limitations of the thermal source.

Most thermal feedback displays employ thermoelectric devices (TEDs) as their thermal source^7,8^. The TED is a heat transfer module for a heater or cooler, which converts electric current into heat flux induced by the Peltier effect. Based on the Peltier effect, if a current passes through the thermoelectric legs in TEDs, heat transfer occurs from one side of the thermoelectric legs to the other such that one side is cold, and the other side is hot. In addition, the direction of heat flux can be switched according to the direction of current; this makes it possible to employ TEDs as heaters or as coolers based on electric control. Owing to this characteristic, TEDs have been utilized in heat transfer systems to realize thermal feedback in wearable devices for VR and AR^9–13^. However, the structure of conventional TEDs incorporates a rigid substrate such as a dielectric ceramic plate. Due to their stiffness, such materials are not suitable for a glove-type device owing to the frequent bending of the hand and unfolding of the fingers. Furthermore, due to the necessity of adaptability to various geometrical forms, research on flexible TEDs has been carried out^14^. The flexible TED developed for an electric power generator based on the Seebeck effect in previous studies^15,16^ exhibits high flexibility and excellent thermal output performance, thereby proving its adaptability to glove-type devices.

**Figure 1:**
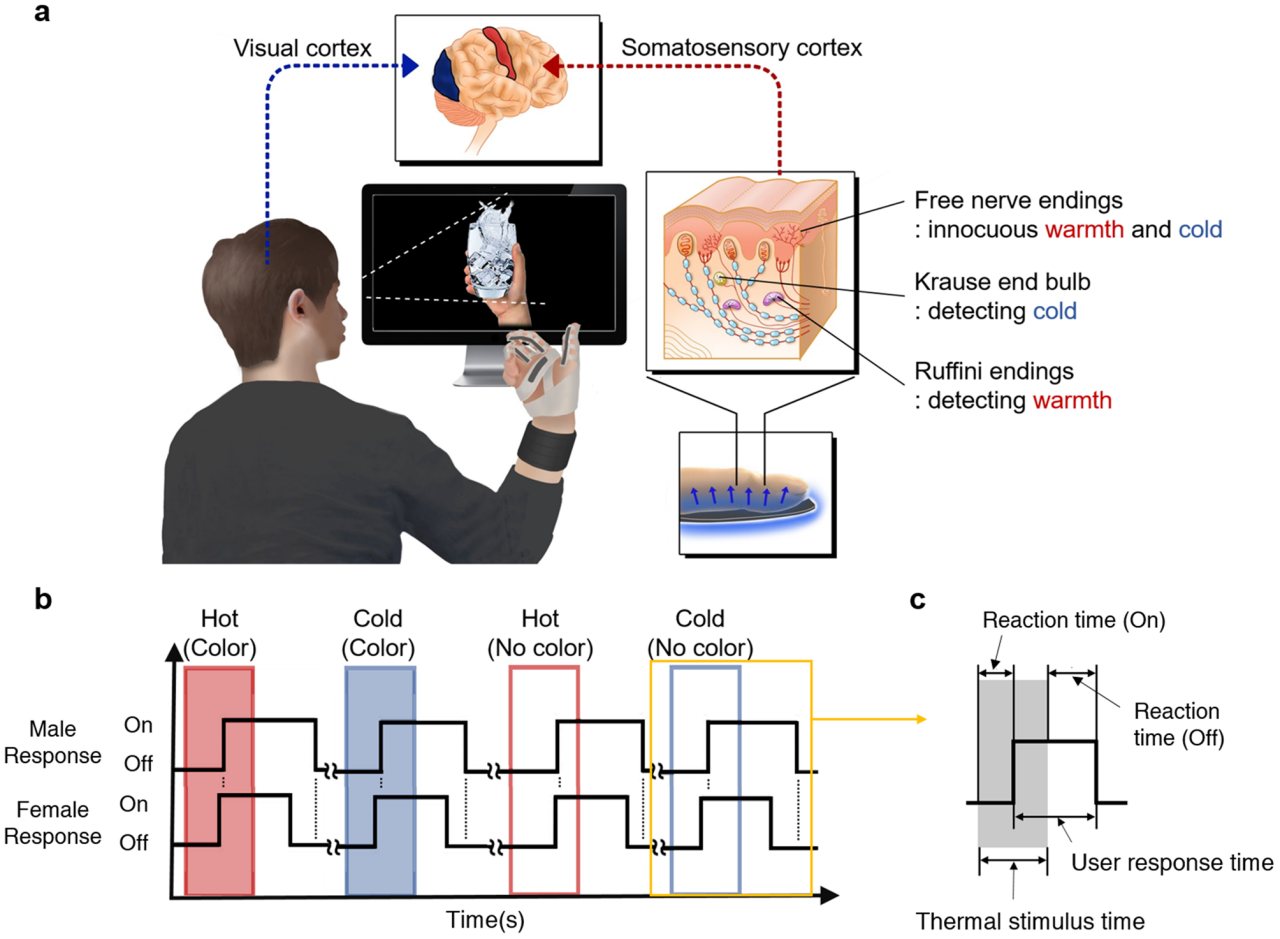
(**a**) Illustration of the operation of thermal display glove system linked to the virtual environment and physiological process. The person wears the proposed thermal display glove and grabs the cold cup in the virtual space. The person receives two different stimuli from the VR: visual and thermal. The brain processes these two external stimulations as if they were from a real situation, and the user can feel realism from the virtual environment. (**b**) Response time to the thermal and color stimuli in the user test. When the VR system showed a colored or non-colored virtual object with a thermal stimulus of a predetermined temperature level for a specific time, the user responded with whether they could feel the thermal stimulus or not. The red boxes show the period for which the system provided a hot virtual object, and the blue boxes show the time in which the system provides a cold virtual object. The solid black lines denote the responses of male and female. The responses of the users were “On” when they were feeling thermal stimulus and “Off” when they were not. The plot was created based on the average response time of the subjects of each gender. (**c**) Definition of reaction time to the thermal stimulus. During the user test, there was a delay between the thermal stimulus and the user response. The reaction times to on/off stimulus were set to the latency between the on/off time of thermal stimulus and user response, respectively.

In this paper, we introduce a novel glove, to interact with a VR environment in real-time, that senses the user’s hand motion and provides feedback through a thermal display that employs a TED (see Fig. 1). The base platform of the glove is the previously developed motion-sensing glove with embedded piezoelectric sensors at the backhand and each finger for measuring the strain of the glove induced by hand motions^17^. The real-time thermal display is realized by using customized flexible TEDs with a suitable form factor that can support finger movement and target minimum curvature radius following the manufacturing process in the previous research^15,16^. In this study, the flexible TEDs are utilized as a thermal display based on the Peltier effect. They are embedded on the front side of the thumb, index finger, middle finger, and upper part of the palm to generate a temperature difference indicating a hot or cold state when the user contacts an object in the VR environment. The contributions of this study to the field of human–computer interactions are as follows.

First, we present a fully untethered thermal display glove that interacts with a VR environment in real-time. The fully untethered and lightweight system enhances the wearability of the device, and it allows the user to move freely in any posture. In addition, the flexible TED can display a wide range of temperature within a few seconds. This range of display temperature allows the user to experience a variety of thermal perception in real life from the VR environment.

Second, we verify the quality of the thermal display glove through a user test, with respect to the realism and presence of the VR environment. In the user test, we follow a questionnaire proposed in a previous study regarding the evaluation of presence in virtual environments using a haptic and thermal displaying vest^18^. Owing to the advantages of wearability and the thermal display performance of the flexible TED, the developed glove improves user immersion in the VR environment. As a result, a majority of the subjects agreed that the stimuli provided by the glove are well-associated with the events in the VR environment.

Finally, using the proposed thermal haptic device, we attempted to investigate the effect of the correlation between object color in VR and thermal stimulation temperature on thermal perception. Previously, Ho studied the impact of the color-temperature correspondences on thermal perception^19^. The experimental results showed that these factors influence response time, that is, the human’s information processing efficiency and thermal perception. However, this previous study was conducted with a hand being placed in a fixed thermal stimulation platform and focused on only two colors (red and blue). Kammermeier et al.^20^ attempted to render the thermal sensation with a glove-type device, but the device was bulky and the sensation was limited in the fingertip. In this study, we utilized the proposed thermal haptic glove with a VR system in an experiment. The experimental conditions included three colors (red, blue, and grey that represent hot, cold, and neutral, respectively) and gender. The experimental results indicated that, based on life experiences with thermal stimuli, people unconsciously expect color-related thermal stimuli in objects of a specific color in VR, and showed that the response time to thermal stimuli varies slightly between males and females.

## Results

### Thermal display glove system

**Figure 2:**
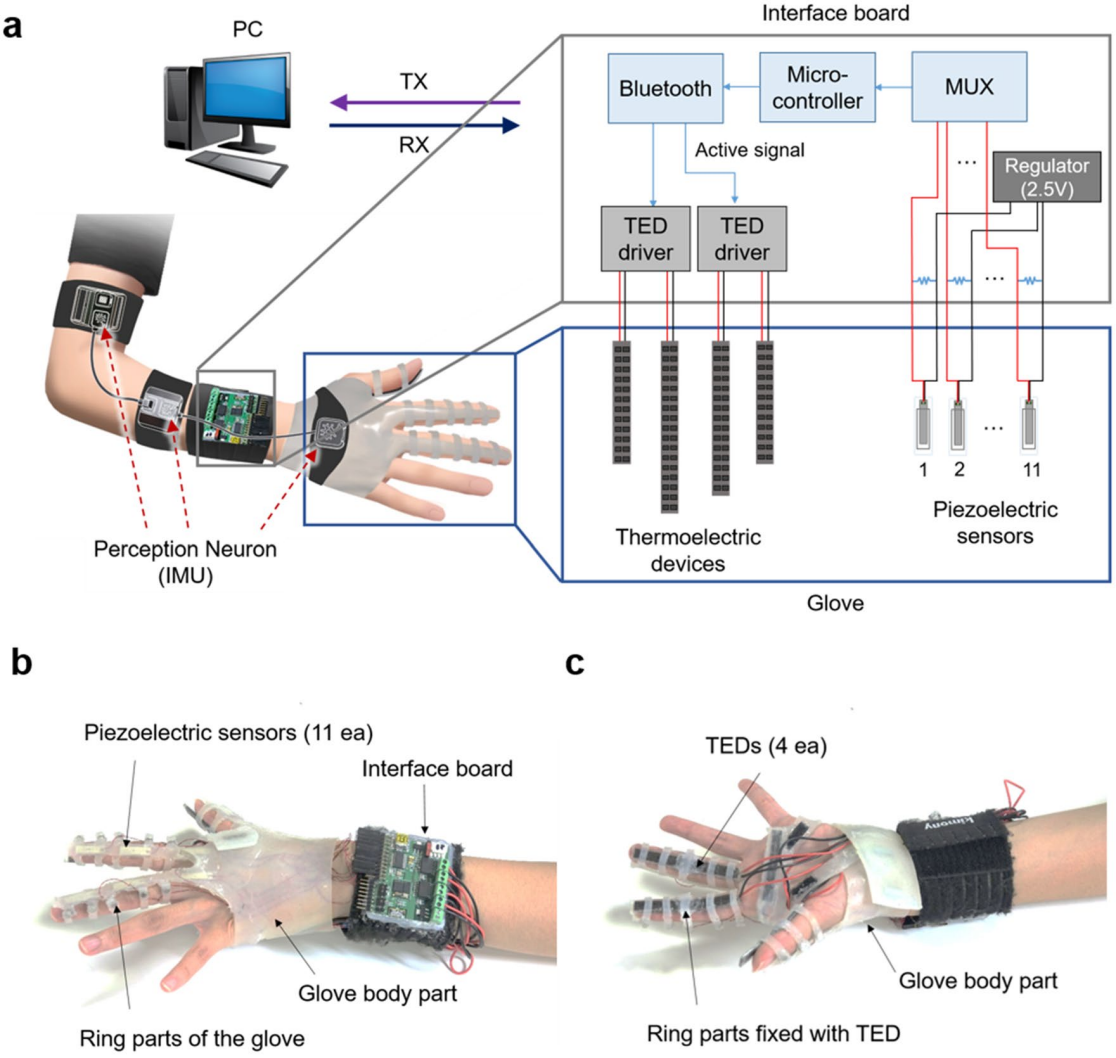
Thermal display glove system. (**a**) Schematic diagram of the thermal display glove system. The system contains the thermal display glove including flexible TEDs, piezoelectric sensors, interface board, and computer software for the virtual environment. Via wireless communication between the interface board and the computer, virtual objects can be interacted with and thermal feedback can be obtained. In addition, a wearable IMU device (Perception Neuron) is used to position the virtual hand. (**b**) The glove on the back, and (**c**) on the palm of the hand.

The thermal display glove system (see Fig. 2) consisted of two main parts: hardware and software. The hardware part included the glove with four flexible TEDs, 11 piezoelectric sensors, and an interface board. The silicone (Ecoflex 00-30) body of the glove was fabricated for installation of the flexible TEDs and piezoelectric sensors. The piezoelectric sensors were aligned with the finger joints of three fingers (thumb, index, and middle finger) and the backside of the hand to track the hand posture^17^. To initialize and stabilize the piezoelectric sensor of the glove, the strain of the sensor must be minimized, and hand should initially be in an open posture like Fig. 2b. The flexible TEDs were attached to the palm as well as the three fingers. The flexible TEDs had a size of (length) 50 mm × (width) 5 mm for the thumb and the palm, (length) 70 mm × (width) 5 mm for the index finger, and (length) 75 mm × (width) 5 mm for the middle finger. The dimensions of flexible TEDs were selected to fit the size of each finger. In addition, flexible silicone fastener rings were secured to the fingers with magnets to ensure that the thermoelectric element and skin are in complete contact. The procedure of wearing the thermal display glove is shown in Movie S1. The interface board was composed of a main microcontroller (ATMEGA328P-AU), a Bluetooth module (F1E22), and two TED driver ICs (TB6612FNG). The analog–digital converter in the microcontroller measured the sensor outputs through a multiplexer and 10 MΩ load resistors, and one driver IC controlled two flexible TEDs. The software part produces the virtual environment and communicates with the interaction board. In the software part, the measured piezoelectric sensor output was used to calculate the joint angles of the actual finger to produce the motion of the virtual hand. Details of the VR program and hand-motion tracking have been presented in previous publications^17,21^. Herein, the thermal feedback is newly incorporated into the VR software. When the virtual hand touches an object with an assigned temperature level, the contact information is transferred to the interface board to operate the TEDs. The TED drivers operate the flexible TEDs on the glove, and the temperatures of the flexible TEDs are changed. Simultaneously, the real hand can feel the thermal stimulus from the virtual environment via the change in temperature of the TEDs. Additionally, we use a wearable IMU device, Perception Neuron, for hand palm positioning in 3D space. The IMU is used on the shoulder, elbow, and wrist.

### Thermal and mechanical characteristics of flexible thermoelectric device

Conventional TEDs are solid-state devices that can convert electrical energy into a large temperature difference across the device through a phenomenon called the Peltier effect. The flexible TED used here is similar to the conventional device but without a ceramic substrate. The fabrication method is described in the “Methods” section. The device figure of merit (ZT) of the flexible TED is 0.74, which is similar to that of a commercial rigid TED (0.78, PTC Ltd.). The proposed TED has a faster response time than that of a conventional TED (Fig. S1). For instance, the conventional device, when the temperature of the hot side is fixed at 20 °C, requires 0.88 s to decrease the temperature by 10 °C when a 4 A input current is applied. Contrarily, the flexible TED requires less than 0.5 s to decrease the temperature by 10 °C at the same current input. This is because the ceramic plates are not attached to the electrodes of the flexible TED.

Flexibility and durability are the most important properties of flexible TEDs for the application in the VR glove. With respect to the flexibility, the major component determining the flexural rigidity of the flexible TED is urethane foam, and its Young’s modulus, measured via a uniaxial tensile test (ASTM D412-A), was 0.53 MPa. Using the modulus and cross-section geometries of flexible TEDs, the flexural rigidity was calculated as 1.767 N mm^2^. Therefore, this low stiffness of flexible TEDs does not interfere with the free motion of a hand wearing the thermal display glove. For the durability, the device should maintain its performance after repeated bending for a bending radius of 10 mm or less; this is the minimal requirement of a roughly grasped hand posture. However, most of the studies on flexible TEDs have not provided data on the durability with respect to repeated bending, even though the devices exhibit flexibility for a very small bending radius. For our device, we conducted a durability test for repeated bending. The test results show that the device maintains the resistance variation within 20% for 800 bends (at a bending radius of 10 mm) and 60 bends (at a bending radius of 5 mm) (Fig. S2a). When the bending radius of the device exceeds 20 mm, the resistance change is insignificant even after the device is bent several thousand times. However, we observe a significant degradation in the performance at the 5 mm bending radius. Hence, we conclude that further research on flexible TEDs is required, which is planned to part of future work.

### Thermal displaying on hand

The flexible TED can display a range of temperatures. When 18.5 W of power was applied to the flexible TED for 2 s, the surface temperature increased up to 70 °C and decreased to 0 °C as shown in Fig. S3a-b. It may be unsafe to apply such rapid thermal change directly to the human hand. To address this issue, two protection methods were applied to the flexible TEDs in the proposed system. First, we attached a 20 μm thick PET film to the top and bottom surfaces of the flexible TEDs to protect users from electric shock and thermal damage. Second, we designed the interface board to limit the maximum electric power applied to the flexible TEDs so that thermal damage to both the users and flexibles TEDs due to drastic temperature changes can be prevented. However, these safeguards diminish the thermal displaying performance of flexible TEDs. To verify the diminished thermal displaying performance of the flexible TED due to insulating PET film and power limited interface board, the temperature change was measured using a temperature sensor (NB-PTCO-153(PT1000)) with the sensing frequency of 2 kHz at two different positions; one position is in the air and the other is on the hand (Fig. S3c-d). The PWM output power of the interface board was 0.452 W. The measured data were filtered with three filters: a low-pass filter to remove the 60 Hz power source noise, a band-stop filter to eliminate the frequency range from 40 to 80 Hz, and another band-stop filter to remove the frequency range from 170 to 190 Hz. Each test was conducted five times and the result was presented in Table S1 and Fig. S3e-j. According to the temperature transition in Figure S3e-h, the insulation effect of the PET film reduced the temperature change by approximately 23% for hot stimuli and 38% for cold stimuli. Furthermore, when the flexible TED was attached to the skin, the temperature change decreased more due to the thermal diffusion from the flexible TED to the skin via conduction (Fig. S3i-j). As a result, the final temperature change was gradual enough for thermal perception but not enough to harm the user. If the input power delivered to the flexible TED is increased, the temperature range would be expanded.

### Thermal interaction experiment between the user and virtual environment

**Figure 3:**
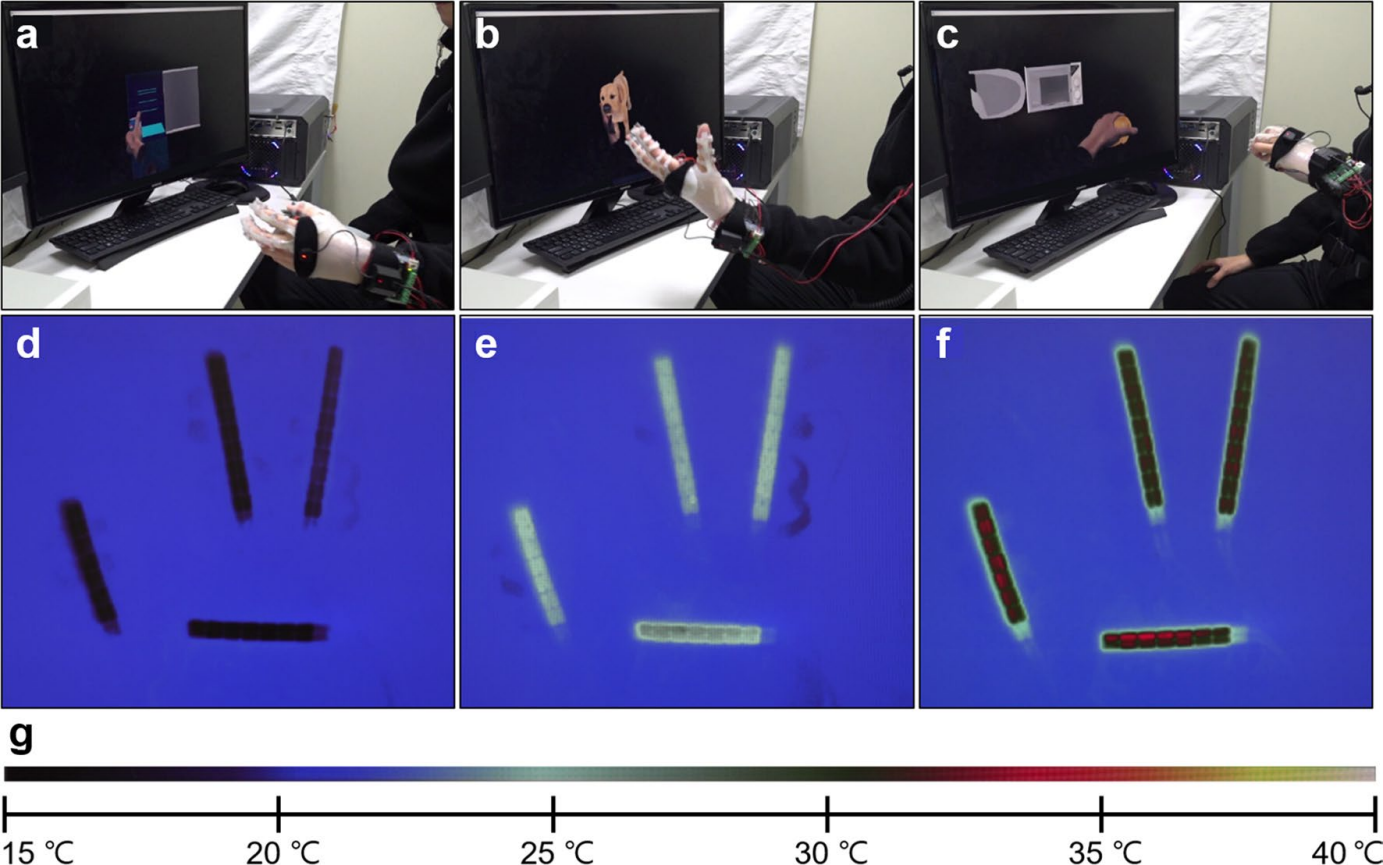
Demonstration of the thermal display glove system. (**a**) Taking a coke can out of a refrigerator, (**b**) petting a dog freely, and (**c**) grasping a chicken in a microwave. (**d**–**f**) show the screen of the thermal imaging camera corresponding to (**a**–**c**). (**g**) Legend of the temperature displayed by the thermal imaging camera. The range of temperature is between 15 °C and 40 °C. The motion of the thermal display glove, the virtual environment, and the real-time thermal feedback were recorded as supplementary movies (Movie S2–S4). In the supplementary movies, two monitors were used; one displayed the virtual reality and the other showed the thermal change of the TEDs in dummy glove (Fig. S4). We used a dummy glove to show thermal change of the TEDs without finger occlusion.

To elucidate the thermal feedback performance of the proposed system, we conducted an experiment where the user interacted with several virtual objects with general temperature expectations. The virtual objects were a coke can in a refrigerator (cold), a dog (warm), and a chicken in a microwave (hot). Specifically, we performed three actions with the virtual objects in the virtual space: (1) taking a canned drink out of a refrigerator (Fig. 3a and Movie S2), (2) freely petting a dog (Fig. 3b and Movie S3), and (3) grasping a chicken in a microwave (Fig. 3c and Movie S4). In these experiments, the temperatures of the flexible TEDs were changed when the virtual hand came in contact with the virtual objects at the designated temperatures. Similarly, the flexible TEDs on the glove were cooled when the user took the can out of the refrigerator, mildly heated to achieve a warm feeling when petting the dog, and heated when the virtual hand grasped the chicken. Initially, the flexible TEDs were at a background temperature of approximately 22 °C. In the cold case, the temperature of the flexible TEDs was reduced to approximately 15 °C during contact with the coke can (Fig. 3d). Moreover, the temperature of the flexible TEDs was changed to 25–28 °C during the interaction with the virtual dog (Fig. 3e). In addition, the temperature of the flexible TEDs increased to approximately 33–35 °C when the user touched the chicken (Fig. 3f). We demonstrated that the proposed system can provide varied temperature changes based on the designated thermal state of the virtual object. During the experiment, the interaction with the cold object decreased the device temperature by approximately 7 °C, the warm object increased the device temperature by approximately 3–6 °C, and the hot virtual object increased the device temperature by approximately 11–13 °C. These three different temperature variations show that the system provides adequate thermal feedback depending on the situation. The differences in the increase in the temperature for the hot and warm objects proves that the proposed system determines the type of thermal change (heating or cooling) and selects the extent of the thermal stimulus. In addition, the motions of the virtual hand were identical to that of the user throughout the experiment (see Movie S2–S4). The user could perform three successful real hand actions, without any obstruction from the equipped components. These results indicate the high flexibility of the device, its ability to withstand various motions of the finger, and the feasibility of real-time interaction between the virtual space and the real world, when using the system. According to previous studies on thermal feedback with heat conduction modeling^8,20,22,23^, it is possible to realize different thermal perceptions via temperature control. If we can control the heat flux precisely and rapidly by adjusting the temperature profile of the TED, the thermal display glove can provide different material sensations from the virtual object even if the shape of the displayed virtual object and the material of the TED attached to the skin remain the same. This will be part of our future work.

### User test of flexible thermoelectric device in virtual reality

The user test was designed to determine if the existence of visual stimulus (color) in VR affects thermal feedback. This test involved 20 participants donning the flexible TED in a VR environment, and the accuracy and reaction time were measured. The participants were divided into two categories based on their gender: male (n = 10) and female (n = 10). We introduced gender classification based on the previous studies that reported different thermal sensitivity according to the gender of the participants^24–27^. The user test was conducted in two stages. For the first stage, the temperature of the virtual object changed to hot or cold as the color of the object changed from gray to red or blue. For the second stage, hot or cold stimulus was provided like in the first stage, but the color of the object remained gray. In both stages, the thermal and color stimuli were provided simultaneously when the virtual hand touched the object. The temperature level (hot (30 °C) or cold (20 °C) as shown in Fig. S3g-h) was assigned to the virtual object randomly during the test to prevent the participants from predicting the temperature level. Before the main test, the participants underwent one preliminary test to adapt themselves to the thermal stimulation. The process of providing the stimuli in each stage is shown in Movie S5 and S6 As shown in Fig. 1b, the participants responded as “*On*” when they started to feel the thermal stimulus and “*Off*” when the stimulus was removed. The reaction time is defined as the interval from the time when thermal stimulation is turned on/off to the time when the user responds by pressing a button based on the stimulation (Fig. 1c). By measuring the reaction time, we verified the effect of the three factors on thermal perception in males and females: (1) type of thermal stimulus (hot/cold), (2) presence of vision (color/no color), and (3) type of response (on/off). By comparing the responses and the defined object temperatures, we can deduce if the users’ response corresponds to the stimulus. The accuracy of the responses is defined as the percentage of the correct answers. The reaction time was determined solely from the correct answers.

The reaction accuracy of each user is shown in Table S2. The results of the accuracy in the first stage, which involved both thermal and color stimuli, indicate that male participants exhibit an accuracy of 93%, and female participants exhibit an accuracy of 95%. In the second stage, which did not involve color stimuli, the male participants exhibited an accuracy of 94%, and the female participants exhibited an accuracy of 88%. The overall accuracy of the system for all participants was 92.5%, which indicates that the participants can perceive thermal feedback greatly. In addition, the average accuracy was 94% when thermal and visual stimulations were both provided, and it is 3% higher than the cases with only thermal stimulation. For female participants, the increase in accuracy with color change compared to that without color change is 7% higher, with on the *p*-value (0.055) being on the edge of significance. On the contrary, male participants exhibited similar accuracies regardless of the existence of a visual stimulus, and the *p*-value for two groups is much higher than 0.05. Therefore, in the case of male participants, the difference in accuracy due to vision is difficult to discuss based on statistical evidence.

**Figure 4:**
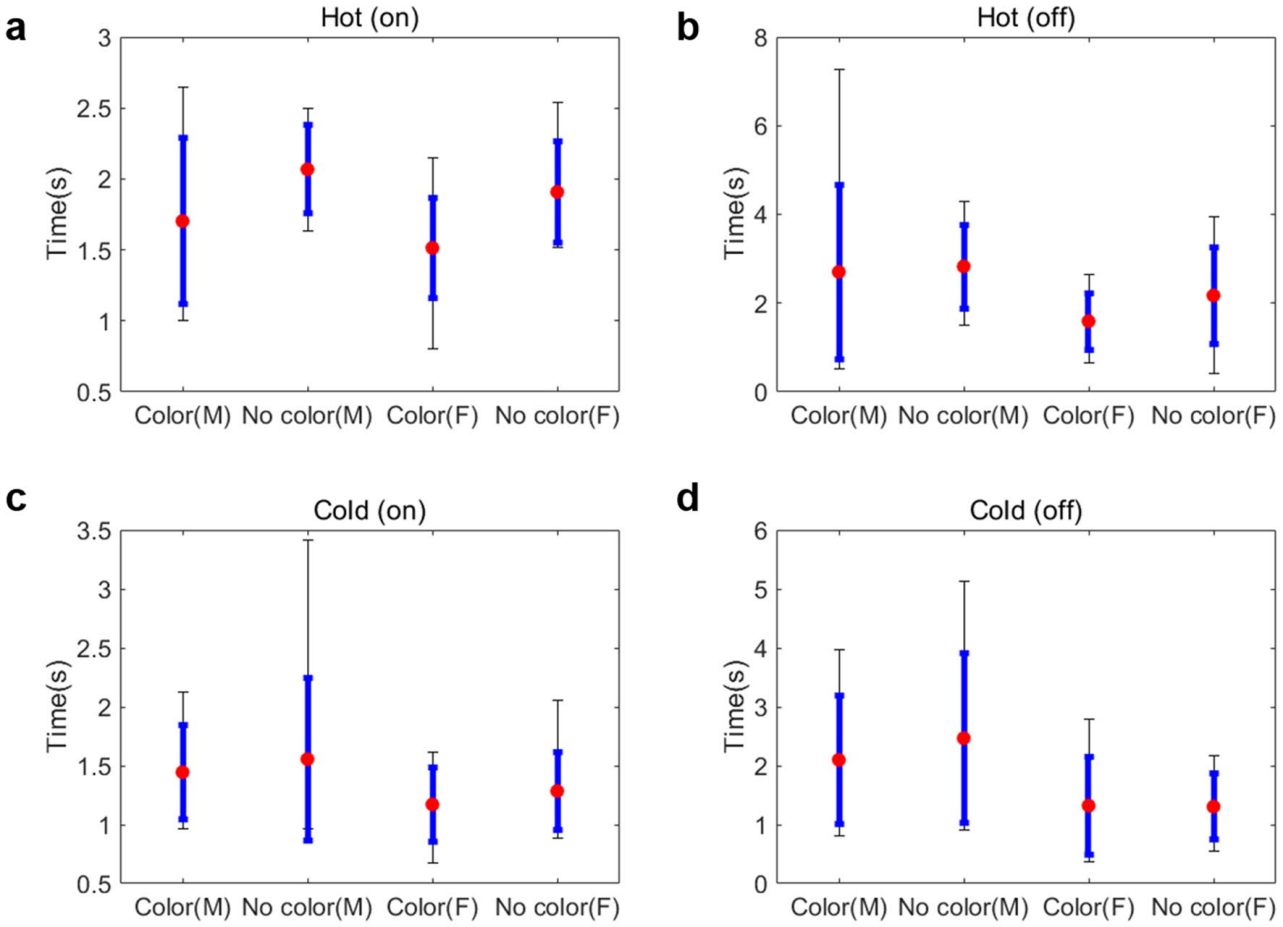
Reaction time in the user test. (**a**) Hot temperature is applied to the virtual object. (**b**) Hot temperature is turned off. (**c**) Cold temperature is applied to the virtual object. (**d**) Cold temperature is turned off. Color denotes thermal and visual stimuli, and No color represents only thermal stimulus. (M) and (F) indicate male and female, respectively. Red dots are the mean values, blue bars are standard deviations, and black bars are the minimum and maximum values.

The reaction times according to different types of stimuli based on temperature, color, and on/off state are shown in Fig. 4. The detailed numeric data are presented in Table S3 and Table S4. The average reaction time of male participants is 2.09 s, and the average reaction time of female participants is 1.54 s. For the stimuli of temperature (hot/cold), color (color/no color), and signal status (on/off), the reaction time of females was less than that of males in all cases. Without gender classification, we can verify the tendency of reaction time for color-temperature cross-modal stimuli in humans. For *Hot/Cold* comparison (Table S4a), the average reaction times of the Hot cases were always higher than those of the *Cold* cases, and *p*-values were always lower than 0.05. The result shows that people react significantly faster to cold stimuli than hot stimuli. For *Color/No color* comparison (Table S4(b)), the average reaction times of the *Color* cases were always shorter than those of the *No color* cases, but the p-value of the *Cold*, *On* case is larger than 0.05. The result indicates that visual stimulus affects the thermal perception significantly in most cases. In the case of signal *On/Off* comparison (Table S4c), the average reaction times of these signal *On* cases were always shorter than those of the *Off* cases, but the *p*-value was larger than 0.05 in the Hot, No color case. This result shows that the thermal perception is faster when the stimulus is provided rather than when it is removed.

### Realism and usefulness of thermal display glove

**Figure 5:**
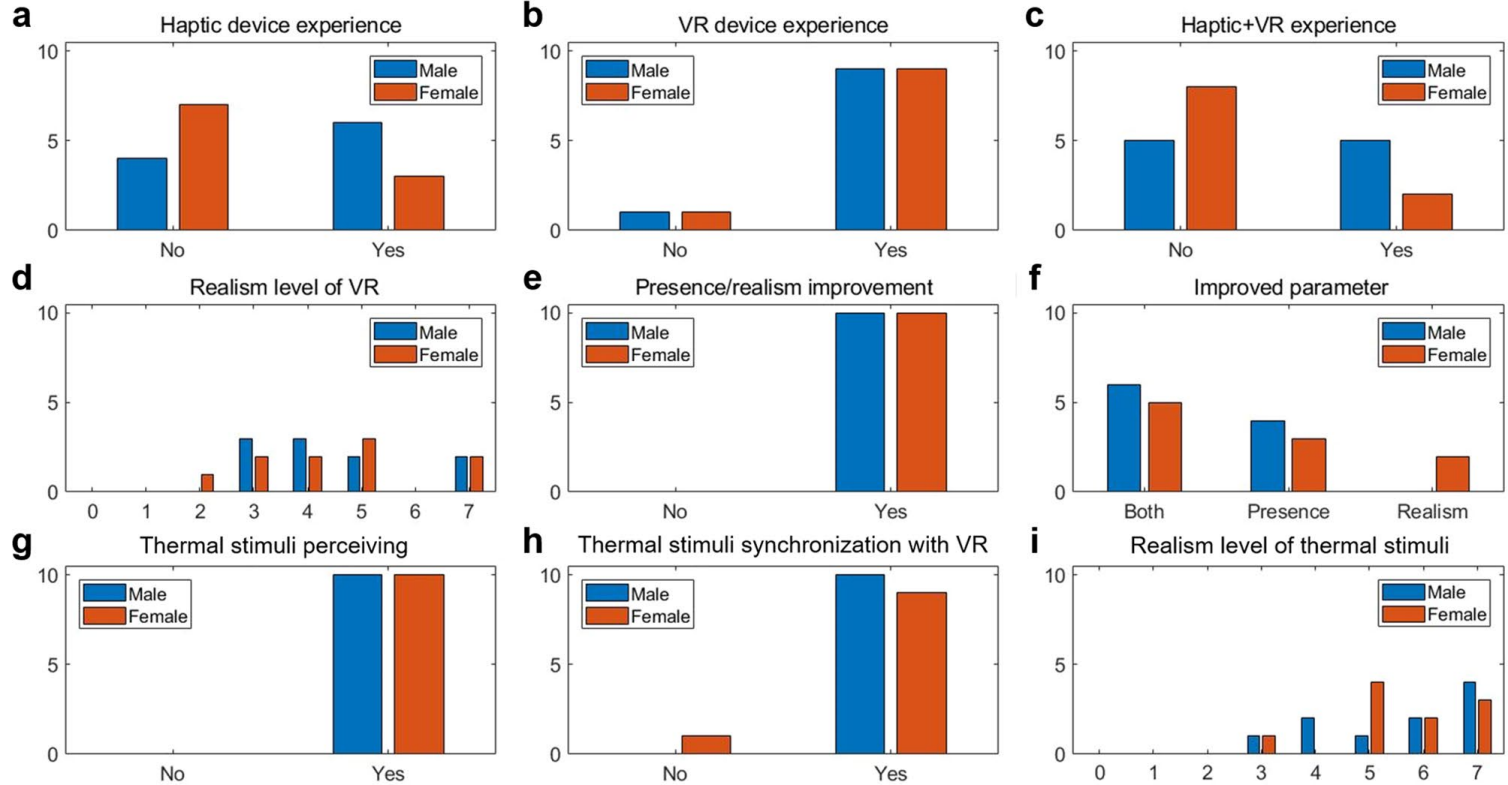
Results of the realism score evaluation. The blue and red colors represent male and female subjects, respectively. Detailed data of the results are provided in Table S7.

We conducted subjective evaluations according to a survey (Table S5 and S6) during the user test. The detailed results are shown in Fig. 5 and Table S7. The participants were divided into three groups based on their response to haptic experiences (Fig. 5a–c): Haptic Experience (*HE*), Technology Experience (*TE*), and Non-Experience (*NE*). This grouping is important because the presence and realism of VR devices can be evaluated differently according to a subject’s level of expertise^18^. We additionally divided the participants into male and female groups to observe the differences in subjective evaluation arising due to gender. The number of participants in each experience group is listed in Table S7. Since the size of each group was small, the statistical significance cannot be adequately generalized from this result.

The scores (Fig. 5d) for the evaluated realism level of the motion sensing device were divided into a range of 3–7 for male participants and 2–7 for female participants. The average value was 4.5 for both male and female participants. This score fell between two realism scales, 4: realistic, non-immersive and 5: realistic and immersive. Therefore, our motion sensing device was evaluated as realistic and partly immersive by the users. With respect to experience, the *NE* male showed the highest response in terms of realism, and the lowest score was given by *HE* males. Female participants gave the highest realism score in the *HE* group and the lowest score in the *NE* group. All participants thought the motion sensing device was useful for improving presence or realism in virtual environments (Fig. 5e). All males thought a motion sensing device was useful for improving presence, and 60% of them thought it was also useful for improving realism. 80% of the female participants responded that the device was useful in terms of presence, and 70% responded that it was useful in terms of realism. (Fig. 5f). The motion sensing device improved presence more than realism for both genders.

Regarding the evaluation of thermal stimuli by the TEDs, all participants indicated that they had perceived thermal stimulus (Fig. 5g). In addition, every participant, except a single *NE* female, responded they could associate thermal stimuli from the thermal display glove with events that happened inside the virtual environment (Fig. 5h). The evaluated scores (Fig. 5i) of the realism levels of thermal stimuli were divided into a range of 3–7 for male and female participants. The total average score was 5.6 for both genders. This score falls between two scales: 5 denotes realistic and immersive, and 6 represents real, with some details not being immersive. This indicates that the thermal stimulus provides users with more realistic and immersive experiences in VR, and these experiences can even feel partly real.

## Discussion

The results from the series of experiments show that the proposed system provides actual thermal feedback on the hands of the users effectively. Through the interactions with various virtual objects, we demonstrated the feasibility of real-time thermal interactions between the virtual space and the real world using the proposed system. In addition, the temperature measurement of the TED in the glove indicates that the generated heat was actually transferred to the user’s hand. The user test shows the performance of the proposed system in two ways, quantitative factors and subjective evaluation.

Moreover, we obtained several important results as follows:

- Visual stimulus helps to recognize thermal feedback more accurately and faster in the virtual environment.
- Sensitivity to the thermal feedback from the virtual environment shows a similar tendency with the actual thermal stimulus.
- The proposed thermal feedback helps to improve user realism in VR.

Regarding the first result, we observed that visual stimulus shows a strong correlation between vision in VR and thermal stimuli with reduced reaction times. Experiments to investigate the influence of color in the perception of thermal stimulus have been conducted in previous studies^28–30^. These studies reported that the human perception of thermal stimulation is influenced by color. Especially, in the study conducted by Ho et al.^30^, the result shows that color-temperature correspondence affects the reaction time required to determine if a thermal stimulus is hot or cold. These previous studies support our result that visual stimulus accompanied by color change affects the thermal perception of a human. As stated in previous publications^30,31^, humans create a cross-modal correspondence between color and temperature in the color-thermal direction.

In most studies that demonstrate the correlation between visual stimulus and thermal perception, the visual stimulus is provided before the thermal stimulus. However, unlike previous studies, the visual stimulus was provided simultaneously with thermal stimulus in our present study. This was possible due to our innovative experimental VR environment. In the real world, the sudden appearance of an object is almost impossible. Thus, a human first perceives the object via vision and then touches it. However, in a VR space, objects can be created suddenly. Therefore, in this study, we provided thermal and vision stimuli simultaneously and observed the feedback of users with and without vision to demonstrate that the created visual stimulus helps recognize thermal feedback more accurately and rapidly in the virtual environment. As indicated by our results, visual stimuli provided with thermal stimuli help the thermal perception of humans in VR environment.

Second, we analyzed our user test results in terms of the reaction time to the thermal feedback. According to the result, the reaction time for thermal feedback was significantly shorter with cold stimulus than hot stimulus. In addition, the responses of females were faster than that of males. This result can be explained by previous studies regarding the variation in thermal sensitivity depending on thermal stimuli and genders^8,24–27,32,33^. In Golja's research^27^, it was assumed that the summation of neural inputs from specific and non-specific cold thermoreceptors rapidly reaches the threshold faster than in the case of a warm sensation. Additionally, the conduction velocity of cold afferent fibers is approximately 10 times higher than that of warm fibers^8^, and the reaction time for the cold stimuli is significantly shorter to warm stimuli^33^. Females are generally more sensitive than males to thermal stimuli^24–27^. Particularly, females have a higher spatial sensitivity for mechanoreception and a greater temporal summation for thermal pain than males^27^. In addition, the epidermis of females is observed to be thinner than that of males^25^. These facts result in higher thermal sensitivity in females. This biological background can explain why, in this study, female participants felt cold stimulation faster and help characterize the relation between gender and reaction time to the thermal stimulus.

According to the third result from the questionnaire about the realism of the proposed system, the realism score of the thermal display glove was higher than that of a motion sensing device only. With respect to the evaluation of only the motion sensing device, the user evaluated the VR system as realistic and partly immersive. Meanwhile, thermal stimuli improved the evaluation score to realistic and immersive, and even caused the VR to feel partly real. The improvement of the evaluation was shown in every group of participants, regardless of haptic and VR experience or gender. Consequently, we verified that thermal stimuli help improve the realism of VR for actual users.

In the evaluation test, an interesting result can be observed from the *NE* users. The response of a female *NE* participant was completely different from those of others. She felt no presence from the motion device and did not associate the VR with thermal stimuli. According to the result from the user test for thermal perception, this participant was less sensitive to thermal stimulus than other female participants were. Her average reaction time for the VR was approximately 2.1 s, which was slower than the average reaction time of other participants (All 1.82 s, female 1.54 s). On the contrary, a male *NE* participant responded to the questions very positively, and his average reaction time was approximately 1.67 s, which was faster than the average reaction time of other participants (all 1.82 s, male 2.09 s). Though the size of each sample is very small, thereby making it difficult to derive statistical significance, there may be a correlation between the sensitivity of perception and score of realism and presence. To verify the correlation, an additional experiment with a large number of *NE* participants is required. This will be addressed in our future work.

Unlike the difference in reaction time, the average evaluation scores with respect to both the motion sensing device and thermal stimuli do not vary with gender. Despite the variation in reaction time based on gender, the participants perceived approximately the same level of realism. Thus, biological characteristics affect sensitivity but not the subjective evaluation of the user.

Despite the interesting experimental results, the proposed system has several limitations that need to be addressed. First, there was a delay from the system operation. According to previous studies on the thermal perception of humans^34,35^, the reaction time to perceive temperature change is 0.89–1 s. In the present study, the average reaction time, which is an average latency between the temperature activation and the user response (see Table S3), was 1.82 s, thus we presume that the latency induced by the thermal display system is approximately 1 s. This latency arose from the limitation of the TED driver in the interface board that has a slew rate to drive current output. If an external driver with large output is used, the TED achieves a shorter system delay.

The second limitation is that the overall accuracy in the task is not closer to 100%. Several factors affect the rate of wrong answers in the user test. One factor could be psychological pressure to respond quickly. The test user could press the wrong response button under some pressure to react as quickly as possible before the following stimulus. Before the test, we notified all participants that their response time would be measured and they must respond as soon as they feel the stimulus. During the test, we observed some cases where the user accidentally pressed the wrong button and notified this to the experiment manager. In this case, the manager checked that their answers were wrong and requested additional responses. Nevertheless, there could be a possibility that participants completed the task without notifying their mistake to the manager, because few responses were recorded faster than the rate at which thermal stimuli were applied. Second, the serially positioned response buttons (“A,” “S,” and “D”) could affect the accuracy. The user has to press the buttons using one hand while the other hand interacts with the thermal display glove, which may make it difficult to press the correct response button.

Finally, the thermal display glove has a limitation in terms of maintaining the feeling of cold. As shown in Fig. S3f, S3h, and S3j, the surface temperature increased in the cooling mode before the TED turned off at 3 s. This is caused by the joule heating of the thermoelectric legs and heat transfer from the other side. In the cooling mode, the duration for which the temperature is maintained on the skin is a function of various variables such as input current level, heat dissipation on the hot side, and ambient temperature. The easiest way to address this limitation is to eliminate the overheating completely by simply lowering the input current with little loss in response time^36^. However, considering the response time, minimum temperature, and overheating, the most effective way to improve cooling performance is to increase the heat dissipation of the hot side. Therefore, an effective solution to dissipate heat from the thermal display glove is required, and this will be addressed in our future work.

## Methods

### Fabrication of thermal display glove

A 3D mold for the thermal display glove was designed using Solidworks (Dassault Systems Solidworks Corp., USA) (see Fig. S5a). For the mold manufacturing, an acrylic board was laser cut. Ecoflex 00-30 silicone solution was poured into the acrylic mold and solidified. In addition, the rings used to fix the fingers and the TED attachment to the glove were made using the same silicone solution. The silicone fastener ring was made by rounding the two ends of a silicone strip with small neodymium magnets. The flexible TEDs for the fingers were attached to a single ring located in the middle of the finger using a silicone adhesive (Sil-Poxy, Smooth-On, Inc., USA). The flexible TED on the palm was attached to the silicone band, which was designed to cover the palm.

The composition of the fabricated piezoelectric sensor is shown in Fig. S5b, and the sensor itself is presented in Fig. S5c. We used polyvinylidene fluoride (PVDF) as a piezoelectric material. The PVDF was produced by Measurement Specialties, Inc. and had a thickness of 28 μm. For the PVDF to endure strain under bending, a polyethylene terephthalate film with a thickness of 100 μm was bonded to the PVDF using 3 M DP460 epoxy. The electric wire with copper tape was attached to the end of piezoelectric film to transmit its voltage output. Finally, 3 M Scotch tape was attached to the top and bottom of the piezoelectric sensor to prevent electric shock damage from the sensor. The fabricated sensors were embedded at the specific locations of the glove (see Fig. S5a). The sensors had three nominal sizes: 20 mm × 5 mm for positions 1–7/10–11, 30 mm × 5 mm for position 8, and 40 mm × 5 mm for position 9.

The size of the interface board was 45 mm × 51 mm. The board was attached to the wrist band. To radiate heat, a flexible heat resistant pad with a thickness of 5 mm was placed between the board and the wrist band. The procedure of wearing the thermal display glove is depicted in Movie S1. The thermal glove was worn for approximately 3 min.

The total weight of the thermal display glove, including the sensors and flexible TEDs, was approximately 105.9 g. The weight of the board and the heat resistant pad fixed on the wrist band was approximately 61.9 g. The weight of the two batteries was approximately 156.2 g. One battery was used for running the microcontroller, and the second battery was used for driving the TEDs.

### Thermoelectric device fabrication

The fabrication of the TED begins with patterning copper electrodes onto the poly-imide substrate. After the Sn_96.5_Ag_3.0_Cu_0.5_ solder paste was printed on the copper patterns, N and P type thermoelectric legs were placed on the solder material; this was followed by a bonding process at 250 °C for 10 min, using a reflow machine. After the bonding process, the empty space between the legs was filled with a proprietary polymer having a significantly low thermal conductivity (0.03 W/(m K))^37^. For the thermoelectric legs, a commercially available material (Rustec LLC) with an area of 1.6 mm^2^ and a height of 2.0 mm was used. The flexible TEDs were fabricated in three sizes: 50 mm × 5 mm, 70 mm × 5 mm, and 75 mm × 5 mm. The measured resistances of the flexible TEDs were 1.7 Ω, 1.9 Ω, and 2.0 Ω for 50, 70, and 75 mm, respectively.

### Measurement of thermal and mechanical characteristics of thermoelectric device

To measure the flexural durability, the TEDs were placed on a plate whose temperature was maintained at 20 °C using a chiller to eliminate heat generated on the hot side of the device. The temperature of the cold side was monitored using a Keithley 2700 multimeter. For ZT values, a Z-meter from RMT Ltd. was used. For the bending test, the flexible TED was located between two blocks with actuators moving in opposite directions, and repeated bending was conducted (Fig. S2b). The bending radius at the center of the device was measured with a laser during the durability measurement.

### Operation of flexible TEDs

The flexible TED with the Peltier effect is controlled by a supplied electric input. When current flows through a junction between two thermoelectric legs in the flexible TED, heat is transferred from one side to the other. Specifically, the maximum display temperature on a specific side of the flexible TED can be considered as the amount of current via PWM control, and the mode change between hot and cold can be achieved by changing the direction of current. For the thermal stimulation in the thermal display glove, four flexible TEDs were embedded on the glove and were controlled by two dual TED drivers on the interface board. A power of 0.452 W (output RMS voltage 0.672 V, current 0.67 A) from a single channel of the TED driver was applied to the flexible TED. The power to operate two TED drivers in the interface board was supplied in two ways: using a rechargeable lithium-ion polymer battery or the DC power supply. In the evaluation test for virtual interaction, we used a battery with a capacity of 3,600 mAh at 7.4 V. If the four TEDs of the thermal display glove are used continuously, the available operation time is approximately 1.5 h. During the long-duration user test, we used a DC power supply instead of the battery to prevent power drop.

### Temperature transfer using the proposed system

In the proposed VR system, we stipulated that each virtual object can have three different temperatures: hot, cold, and warm. These temperature level can be realized by controlling the quantity and the direction of the current flow of the TED driver. When a virtual finger touched a hot object, the maximum current from its TED driver flowed in the direction of the generated heat. Conversely, in the case of touching a cold object, the maximum current flowed in the opposite direction to take heat away from the finger. When the user touched a warm virtual object, only approximately one-fifth of the maximum current flowed to the TEDs in the direction of heat generation. Additionally, the proposed system was designed to work independently on the fingers, which means each TED operation was determined by the contact of each virtual finger.

### Design of user test

The design of the user test included participant characteristics, experimental procedure, data acquirement, and a questionnaire. Twenty healthy adults including 10 females (age: 24.7(mean) ± 2.1(SD) years) and 10 males (age: 25.1(mean) ± 1.7(SD) years) participated in the user test. To reduce the effect of users’ age on the thermal feedback^38^, the range of age of the participants was limited. Participants were researchers or college students and had no experience in thermal feedback with VR. This user test was approved by the Institutional Review Board in KIST (KIST2019-035), and the experiments involving humans were conducted in accordance with the ethical standards in the 1964 Declaration of Helsinki. We obtained informed consent from every participant prior to their enrollment in the study. Specific informed consent to the publication of identified information/videos/images in an online open access publication was obtained from a study participant. The room for the user test was isolated to achieve a steady state air condition. The air temperature of the room was maintained at 22 °C. During the test, only a single participant and two experiment managers were presented in the room. The participants sat on a chair in front of the monitor and donned the Perception Neuron device and the thermal display glove. Prior to the user test, hot and cold stimuli were applied to the glove once, respectively, to ensure that the user was able to perceive the thermal change in the glove. The user test was divided into two stages: with visual stimuli and without visual stimuli. For the visual stimulus, we designated that red represented hot and blue denoted cold. In the first stage with the visual stimulus, the virtual object was initially not assigned a temperature. The user test began after the participants came in contact with the virtual object. After a few seconds, the virtual object randomly changed into a hot or cold object, and the thermal stimulus and associated color were applied. The thermal stimulus was applied for 2.75(average) ± 0.25(range) s, and the next stimulus followed after 9(average) ± 1(range) s. This process was defined as a single step, and 10 steps were repeated in the first stage. The interval times were randomly determined in the range ± 0.25 s and ± 1 s. In this stage, the initial color of the virtual object was gray, and it was changed to red for hot and blue for cold. In the second stage without the visual stimulus, 10 steps were repeated as described in the first stage. However, the color of the virtual object was always fixed to gray. Example videos of each stage are shown in Movies S5 and S6.

During the user test, the computer program recorded the temperature change of the virtual object and the user response in milliseconds. Specifically, when the user pressed the buttons, the user response was recorded. For each stage, the users were prompted to press one of three response buttons (“A,” “S,” and “D”) when they started to feel hot/cold sensations on their hand or when they were unable to feel the thermal stimulus. When they felt a hot stimulus, they pressed “A.” In case they felt cold, the users pressed “S.” When the stimulus was removed and they felt no temperature difference, they pressed “D.” Examples of the recorded signal and the response of the users are illustrated in Fig. 1b.

After the user test was completed, the participants were asked to answer the questionnaire. We used a questionnaire similar to that used in a previous study^18^ to evaluate the haptic VR device. The questionnaire was used for two purposes: classifying the participants according to their haptic and VR experiences and analyzing the realism and the effectiveness of the proposed thermal display glove. Our questionnaire included nine questions. Details regarding the questions and the available options are stated in Table S5 and Table S6.

## Supplementary information

Supplementary Information 1.

Supplementary Information 2.

Supplementary Information 3.

Supplementary Information 4.

Supplementary Information 5.

Supplementary Information 6.

Supplementary Information 7.
