# Supplementary material for: Thermal display glove for interacting with virtual reality
Source: Sci Rep. 2020 Jul 9;10:11403. doi: 10.1038/s41598-020-68362-y (PMC7347636; doi:10.1038/s41598-020-68362-y)
Supplement: Supplementary file 7 — Supplementary Information 7. [file 41598_2020_68362_MOESM7_ESM.pdf]

Thermal Display Glove for Interacting with Virtual Reality

Seung-Won Kim[1]*, Sung Hee Kim[2]*, Choong Sun Kim[3], Kyoungsoo Yi[4], Jun-Sik Kim[2], Byung Jin Cho[3,5]†, and Youngsu Cha[2]†

[1] *Center for Medical Robotics, Korea Institute of Science and Technology (KIST), Seoul 02792, Republic of Korea.*

[2] *Center for Intelligent & Interactive Robotics, Korea Institute of Science and Technology (KIST), Seongbuk-gu, Seoul 02792, Republic of Korea.*

[3] *School of Electrical Engineering, Korea Advanced Institute of Science and Technology (KAIST), Daejeon 34141, Republic of Korea.*

[4] *TEGway Co. Ltd., Daejeon 34325, Republic of Korea.*

[5] *Engineering Research Center (ERC) for Flexible Thermoelectric Device Technology, Daejeon 34141, Republic of Korea.*

*These authors contributed equally to this work.

†Corresponding author. Email: elebjcho81@kaist.ac.kr(B.J.C.), givemong@kist.re.kr(Y.C.)

SUPPLEMENTARY MATERIALS

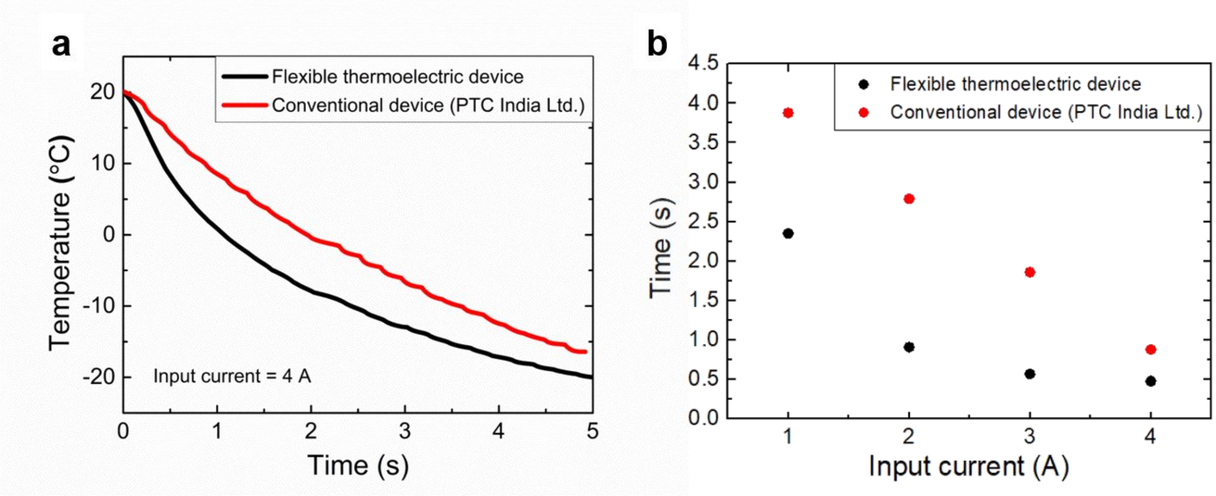

Figure S1: Response time of the thermoelectric devices. (a) Temperature vs. time at the input current of 4 A. Black lines represent the response time of the flexible thermoelectric device, and red lines represent that of the conventional thermoelectric device (PTC Ltd.) (b) Time to decrease the temperature by 10 °C with different input currents.

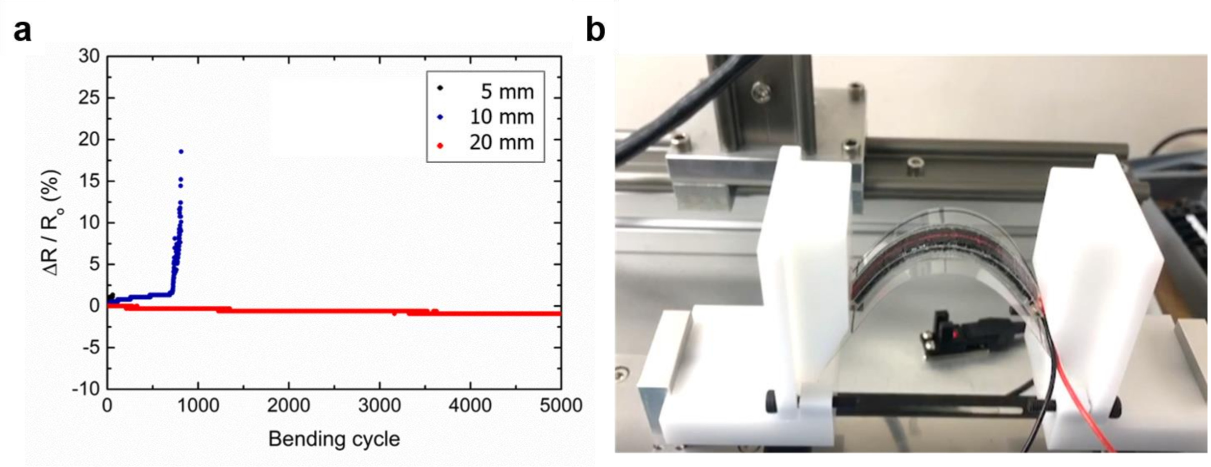

Figure S2: (a) Black, blue, and red dots represent the test results with 5 mm, 10 mm, and 20 mm of bending radius, respectively. Normally, in the bending test, the resistance of a flexible TED gradually increases and eventually break down (blue line). However, in case of the bending radius of 5 mm and 10 mm, the devices were suddenly broken without a significant resistance change because urethane rubber (Clear Flex 50, Smooth-On Inc.) which seals the edge of the device was fractured first, and a large stress was applied to the device. If edge is sealed with a sufficiently flexible rubber, the durability of the flexible TED would be further improved. (b) Experiment setup for the bending test.

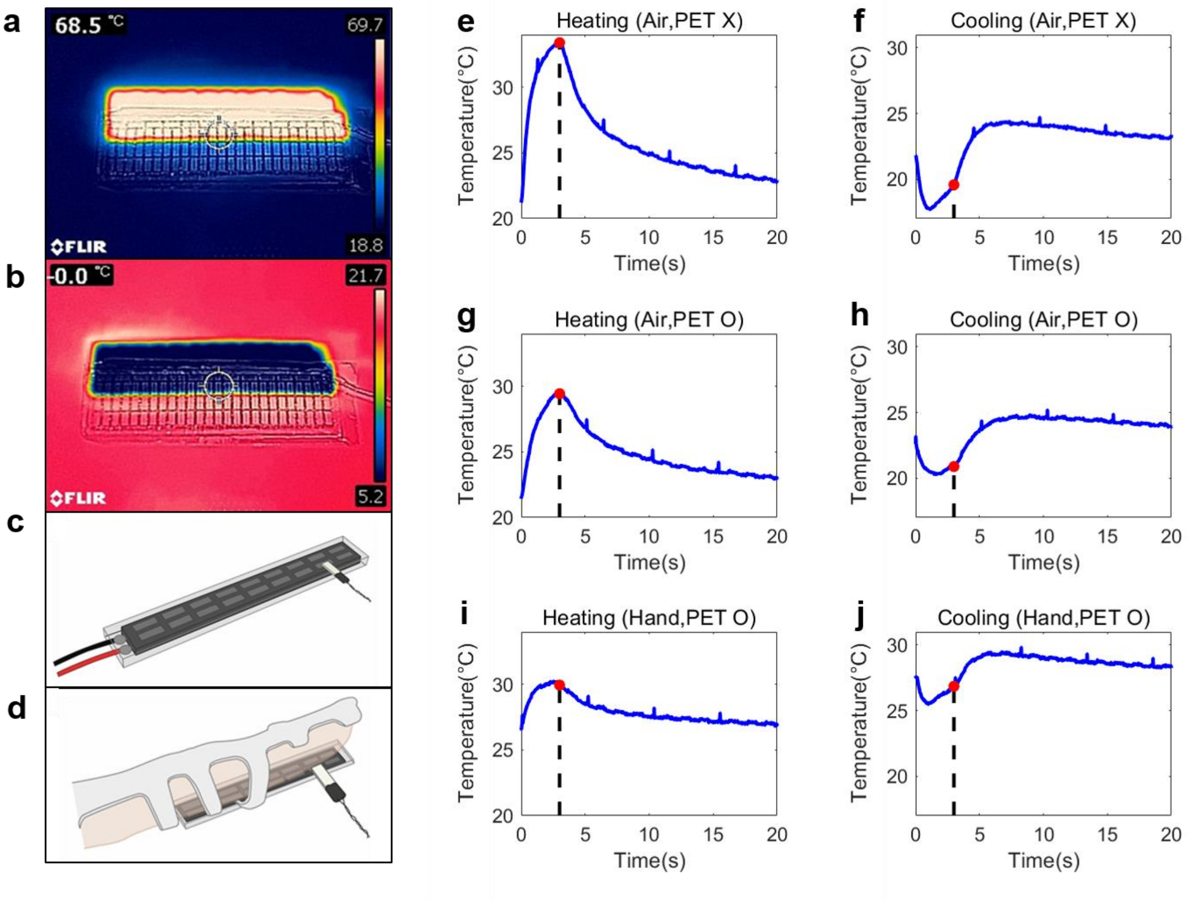

Figure S3: Temperature transition of flexible TED. The surface temperature of flexible TED in (a) hot mode and (b) cold mode was measured by using the thermal imaging camera (FLIR C2). The images were captured when the input power of 18.5 W from an external power supply was delivered to the flexible TED for 2 seconds. Temperature transition of flexible TED activating under safety mode by the interface board was measured at two different positions: (c) in air and (d) on hand. The position of thermocouple was fixed at the right one of the second column from the one end of flexible TED. (e)-(j) show temperature transition of the flexible TED in 6 different conditions for 20 s: (e) heating in air without PET film, (f) cooling in air without PET film, (g) heating in air with PET film, (h) cooling in air with PET film, (i) heating on hand with PET film, and (j) cooling on hand with PET film. The red point in (e)-(j) indicates the time at 3 s when the thermal stimulus turned off.

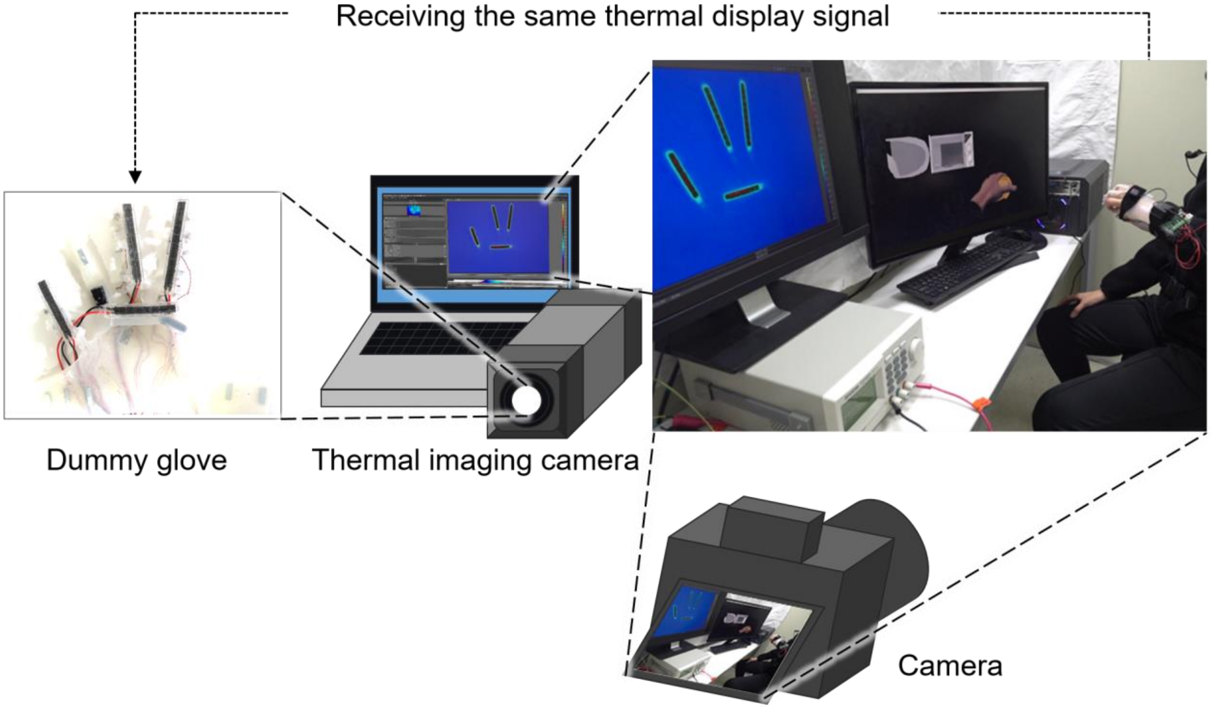

Figure S4: Recording condition of the experiment. We recorded the movement of the thermal display glove, the virtual environment, and the real-time feedback of the system. Also, we added a dummy glove to show the thermal change of the flexible TEDs. The dummy glove is the same as the proposed glove but not worn on the hand. It received the same signal with the main glove, and we observed it by the thermal imaging camera. A single monitor shows the virtual environment implemented by the proposed system, and the other monitor projects the real-time image of the thermal imaging camera.

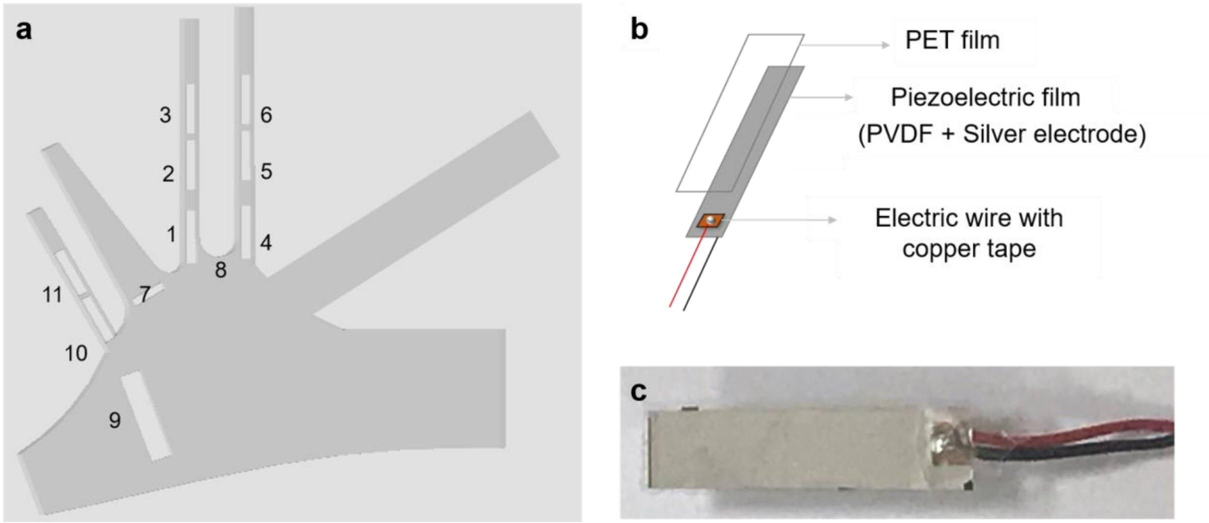

Figure S5: Mold for glove and piezoelectric sensor. (a) 3D shape mold for glove designed by CAD tool with sensor positions. (b) Structure of the piezoelectric sensor. (c) Picture of the fabricated piezoelectric sensor.

Table S1. Temperature change of flexible TED on hand and in air.

Condition		1st [℃]	2nd [℃]	3rd [℃]	4th [℃]	5th [℃]	Average [℃]
Air (PET X)	Hot	12.63	11.88	11.38	12.22	10.66	11.75
	Cold	-3.98	-3.88	-4.13	-3.91	-3.65	-3.91
Air (PET O)	Hot	9.00	8.04	9.60	7.84	10.55	9.01
	Cold	-2.31	-2.37	-2.26	-2.51	-2.76	-2.44
Hand (PET O)	Hot	4.67	4.53	4.14	3.95	3.63	4.18
	Cold	-2.10	-2.06	-2.01	-1.74	-2.15	-2.01

Note: The difference of temperature, T_d, is defined as $T_d = T_p - T_i$, where T_p is the peak value and T_i is the initial ambient temperature (22-23 ^oC). The peak value was selected as the highest temperature when the device was heated and the lowest value when the device was cooled.

Table S2. Reaction accuracy of the individual user

User		U1	U2	U3	U4	U5	U6	U7	U8	U9	U10	Average
Male ($N = 10$)	Color [%]	100	90	100	100	100	80	100	90	80	90	93
	No color [%]	90	100	90	100	80	100	100	90	90	100	94
	Average [%]	95	95	95	100	90	90	100	90	85	95	93.5
	Color-No color difference [%]				1			p-value			> 0.05	
Female ($N = 10$)	Color [%]	90	100	100	100	100	90	100	90	80	100	95
	No color [%]	90	90	80	90	80	70	100	90	100	90	88
	Average [%]	90	95	90	95	90	80	100	90	90	95	91.5
	Color-No color difference [%]				7			p-value			0.055	
Average (All, $N = 20$) [%]					92.5							

* The p-value was calculated by one-sided paired sample t-test.

Table S3. Reaction time at the user test (Average [s] ± S.D [s])

Condition		Male ($N = 10$)		Female ($N = 10$)		Color ($N = 20$)	No color ($N = 20$)	Average ($N = 20$)	
		Color	No color	Color	No color				
Hot	On	1.76 ± 0.65	2.05 ± 0.45	1.51 ± 0.51	1.98 ± 0.49	1.64 ± 0.60	2.02 ± 0.47	1.83 ± 0.57	2.05 ± 1.07
	Off	2.43 ± 1.79	2.65 ± 1.23	1.58 ± 0.77	2.38 ± 1.26	2.02 ± 1.44	2.52 ± 1.24	2.27 ± 1.37	
Cold	On	1.41 ± 0.47	1.56 ± 0.72	1.18 ± 0.37	1.29 ± 0.41	1.29 ± 0.44	1.43 ± 0.6	1.36 ± 0.52	1.60 ± 1.11
	Off	2.15 ± 1.49	2.67 ± 1.79	1.32 ± 1.04	1.27 ± 0.72	1.72 ± 1.33	1.98 ± 1.54	1.85 ± 1.44	
On		1.58 ± 0.59	1.81 ± 0.65	1.33 ± 0.47	1.62 ± 0.57	1.46 ± 0.55	1.72 ± 0.61	1.59 ± 0.59	
Off		2.29 ± 1.64	2.66 ± 1.53	1.44 ± 0.93	1.8 ± 1.15	1.86 ± 1.39	2.25 ± 1.42	2.05 ± 1.42	
Average		1.94 ± 1.28	2.23 ± 1.25	1.39 ± 0.74	1.71 ± 0.91	1.66 ± 1.08	1.98 ± 1.13	1.82 ± 1.11	
		2.09 ± 1.27		1.54 ± 0.84					

Table S4. Comparison of the difference of reaction time for various stimuli

(a) Hot / Cold (T_{Hot} - T_{Cold})	Color		No color	
	On	Off	On	Off
*Average reaction time difference [s]	0.31	0.34	0.79	0.34
P-value	0.0033	0.0379	7.62E-10	0.0343
**(b) Color / Non-color** (T_{Color} - $T_{No\text{-}color}$)	**Hot**		**Cold**	
	**On**	**Off**	**On**	**Off**
**Average reaction time difference [s]	0.54	0.35	0.05	0.34
P-value	1.28E-05	0.0278	p > 0.05	0.0374
**(c) On / Off** (T_{Off} - T_{On})	**Color**		**No color**	
	**Hot**	**Cold**	**Hot**	**Cold**
***Average reaction time difference [s]	0.31	0.28	0.13	0.57
p-value	0.0309	0.0260	p > 0.05	0.0005

* The average reaction time for comparing the hot and cold reaction time is the reaction time at cold subtracted from the reaction time at hot.
** The average reaction time for comparing the color and non-color reaction time is the reaction time at non-color subtracted from the reaction time at color.
*** The average reaction time for comparing the on and off reaction time is the reaction time at on state subtracted from the reaction time at off state.
**** All p-values in the data were calculated by the one-sided two samples t-test. If the variance of the two samples is the same, we used a pooled-variance t-test and used separate-variance t-test if not.

Table S5. Evaluation questionnaire in survey

Block	Questions
Classification of participants	1.1 Have you ever used any haptic device?<br>1.2 Have you ever used any Virtual Reality (VR) device?<br>1.3 Have you ever worked with haptics and VR devices together?
Motion-sensing device contribution to realism or presence	2.1 Assign score to the realism level of your virtual experience by using a motion-sensing device.<br>2.2 Do you think a motion-sensing device could be useful to improve presence and/or realism in virtual environments? (y/n)<br>2.3 What parameters? Presence or realism?
Evaluation of thermal stimuli by a thermal display glove	3.1 Have you perceived any thermal stimulus?<br>3.2 Could you associate those stimuli with events that happened inside the virtual environment? (y/n)<br>3.3 Assign score to the realism level of the sensation you perceived.

Table S6. Scale for realism evaluation

Score	Perception
0	Not realistic, unnatural
1	Poorly credible
2	Artificial, non-immersive
3	Artificial but immersive
4	Realistic, non-immersive
5	Realistic and immersive
6	Real, some details are not immersive
7	Totally real

Table S7. Realism score evaluation

Group		Motion sensing device				Thermal stimuli		
		Usefulness	Presence	Realism	Score	Perception	Association	Score
Male	All (10)	100%	100%	60%	4.5 ± 1.51	100%	100%	5.6 ± 1.51
	HE (5)	100%	100%	40%	3.8 ± 0.84	100%	100%	5.6 ± 1.14
	TE (4)	100%	100%	75%	4.75 ± 1.71	100%	100%	5.25 ± 2.06
	NE (1)	100%	100%	100%	7	100%	100%	7
Female	All (10)	100%	80%	70%	4.5 ± 1.65	100%	90%	5.6 ± 1.26
	HE (2)	100%	100%	50%	5.5 ± 2.12	100%	100%	6 ± 1.41
	TE (7)	100%	86%	71%	4.43 ± 1.62	100%	100%	5.57 ± 1.4
	NE (1)	100%	0%	100%	3	100%	0%	5

Note: The abbreviations in the group indicate the categories that *All* is the user who experiences both haptic and virtual reality technologies, *HE* is the user who experience haptic only, *TE* is the user who experience virtual reality only, and *NE* is the user who has not experienced both technologies. The number inside the parenthesis after categories is the number of participants who belongs to the category. The score is average ± S.D.
